# Central Auditory Pathway Dysfunction in Type 2 Diabetes Mellitus: A Narrative Review of Auditory Brainstem Response Alterations

**DOI:** 10.7759/cureus.115327

**Published:** 2026-08-27

**Authors:** Adil Abbass, Sumaiya Waheed, Vishavdeep Kaur, Ankalayya Bobbara, Aaseem Anand

**Affiliations:** 1 Physiology, Maharishi Markandeshwar Institute of Medical Science and Research, Maharishi Markandeshwar (Deemed to be University), Ambala, IND; 2 Medicine, Maharishi Markandeshwar Institute of Medical Science and Research, Maharishi Markandeshwar (Deemed to be University), Ambala, IND

**Keywords:** auditory brainstem response, auditory pathway dysfunction, central diabetic neuropathy, glycaemic control, type 2 diabetes mellitus

## Abstract

Type 2 diabetes mellitus (T2DM) is increasingly recognized as a disorder affecting both the peripheral and central nervous systems. Although diabetic neuropathy has traditionally been associated with peripheral nerve damage, accumulating evidence suggests that central auditory pathways are also vulnerable to chronic hyperglycaemia. This narrative review synthesizes current evidence regarding auditory brainstem response (ABR) abnormalities in adults with T2DM, with emphasis on their pathophysiological basis, characteristic electrophysiological alterations, clinical associations, and potential diagnostic significance. A structured search of PubMed/MEDLINE, Scopus, and Google Scholar identified 10 observational studies published between 2000 and 2024 that evaluated ABR parameters in adults with T2DM. Across the included studies, the most consistent findings were prolonged wave III and wave V absolute latencies together with extended interpeak intervals (I-III, III-V, and I-V), while wave I latency remained normal in most patients, indicating predominant central auditory pathway dysfunction. A smaller proportion of studies demonstrated additional peripheral auditory nerve involvement, suggesting mixed neuropathic changes. Higher glycated haemoglobin (HbA1c) levels and longer duration of diabetes were generally associated with greater ABR abnormalities, supporting the role of cumulative metabolic injury in central neural conduction delay. Despite methodological heterogeneity and predominantly cross-sectional study designs, the evidence consistently indicates that the auditory brainstem is an early target of diabetes-related neural injury. ABR represents a non-invasive and reproducible tool with potential utility for detecting subclinical central diabetic neuropathy before overt auditory impairment becomes clinically evident. Further multicentre longitudinal studies using standardized ABR protocols are required to validate its diagnostic and prognostic value in routine clinical practice.

## Introduction and background

Type 2 diabetes mellitus (T2DM) constitutes a global metabolic disorder of escalating prevalence, with an estimated 537 million adults affected in 2021 and projections exceeding 780 million by 2045 [1]. Microvascular complications, such as retinopathy, nephropathy, and neuropathy, account for a substantial proportion of the disease burden. Among these, diabetic neuropathy is the most prevalent and clinically heterogeneous, manifesting as sensory loss, neuropathic pain, and foot ulceration that collectively impair quality of life [2]. While the peripheral nervous system has long been the central focus, neurological involvement in T2DM extends beyond peripheral nerves.

Diabetic neuropathy is no longer viewed solely as a length-dependent peripheral nerve disorder; evidence from neuroimaging and electrophysiology has firmly established central nervous system (CNS) involvement. The concept of central diabetic neuropathy has gained traction as structural and functional alterations are demonstrated in the spinal cord, brainstem, and cortex [3]. Sustained hyperglycaemia drives oxidative stress, advanced glycation end-product formation, and microvascular injury, which in turn provoke neuroinflammation and demyelination in both peripheral and central neural structures [4,5]. These pathological cascades compromise axonal integrity and synaptic transmission in metabolically active regions, including brainstem nuclei. The auditory brainstem, with its precisely organized relay stations, thus represents an ideal system for assessing central neural conduction deficits attributable to diabetes. Chronic hyperglycaemia is associated with oxidative stress, advanced glycation, microvascular dysfunction, and metabolic injury, all of which are implicated in diabetic neural complications [4,5]. These processes may contribute to impaired neural conduction and tissue injury within the auditory system. However, the extent to which these mechanisms directly affect the human auditory brainstem remains incompletely established. Evidence implicating oxidative stress, microvascular injury, inflammation, mitochondrial dysfunction, and neuropathic mechanisms in diabetic auditory pathology supports the biological plausibility of diabetes-related auditory dysfunction [5,6].

The central auditory pathway ascends from the cochlear nerve through the cochlear nucleus, superior olivary complex, lateral lemniscus, and inferior colliculus to the medial geniculate body and auditory cortex. Brainstem auditory nuclei maintain high metabolic rates and are particularly vulnerable to ischaemic, metabolic, and inflammatory insults [3,6]. The auditory brainstem response (ABR) is a far-field electrophysiological recording that captures synchronised neural activity along the auditory pathway in response to an acoustic stimulus [7,8]. The ABR waveform comprises several transient peaks within approximately 10 milliseconds of stimulus presentation. Rather than reflecting activity from single anatomical structures, individual waves represent activity from distributed neural generators at various levels of the auditory pathway. Wave I is primarily associated with the distal auditory nerve, while later waves, particularly waves III and V, originate from multiple neural generators within the brainstem [7,8]. ABR parameters are typically described using absolute wave latencies, defined as the time from stimulus onset to the occurrence of a specific wave, and interpeak intervals, defined as the latency difference between two waves. Prolongation of interpeak intervals, such as I-III, III-V, and I-V, may indicate delayed neural conduction along the corresponding segments of the auditory pathway. However, these measures should not be interpreted as precise anatomical localisation of a lesion. A relatively preserved wave I with prolonged later waves or interpeak intervals may indicate predominantly central auditory conduction delay, whereas abnormalities involving wave I may suggest additional involvement of the peripheral auditory pathway [7-9].

Clinical studies in adults with T2DM have documented prolonged absolute latencies of waves III and V and extended I-III, III-V, and I-V interpeak intervals, often with normal wave I latency, suggesting a predominantly central pattern of dysfunction [10,11]. Some investigations, however, have also reported delayed wave I, indicating mixed peripheral and central involvement [12]. Prolongations of wave V and interpeak latencies correlate with higher glycated haemoglobin (HbA1c) levels and longer disease duration, supporting a link between cumulative hyperglycaemic exposure and progressive auditory brainstem dysfunction [13]. Yet the findings remain inconsistent. Heterogeneous study populations, variations in disease duration and glycaemic control, the presence of coexisting peripheral neuropathy, and differences in ABR recording parameters, such as stimulus type, rate, and intensity, all contribute to conflicting results and impede direct cross-study comparisons [10-13].

Previous evidence syntheses have established an association between type 2 diabetes and auditory dysfunction, although these studies have generally addressed broader research questions than those considered here. Gupta et al. conducted a systematic review and meta-analysis focused on ABR findings in type 2 diabetes [11]. Subsequently, Akinpelu et al. performed a systematic review evaluating hearing function in individuals with type 2 diabetes, reporting significantly prolonged ABR wave V latencies and a higher prevalence of hearing loss [12]. While these reviews provide important evidence that diabetes may affect auditory function, the literature remains heterogeneous regarding ABR waveform abnormalities, interpeak intervals, hearing status, glycaemic control, and the distinction between peripheral and central electrophysiological patterns.

This narrative review focuses on interpreting ABR abnormalities in adults with type 2 diabetes. Instead of evaluating hearing impairment as a broad clinical outcome, it synthesises reported patterns of absolute wave latencies and interpeak intervals, assesses whether findings align with peripheral, central, or mixed auditory pathway involvement, and evaluates reported relationships with glycaemic control and diabetes duration.

The review also considers methodological factors that may account for differences between studies and critically examines whether ABR can currently be regarded as a clinically useful marker of subclinical central auditory dysfunction. This narrower electrophysiological focus is intended to complement, rather than duplicate, the conclusions of previous broader systematic reviews.

## Review

Methodology

This narrative review was undertaken to critically synthesise published evidence concerning ABR abnormalities in adults with T2DM. The review focuses on absolute wave latencies, interpeak intervals, possible central versus peripheral auditory pathway involvement, relationships with glycaemic control and diabetes duration, underlying pathophysiological mechanisms, methodological limitations of existing studies, and potential clinical implications. The review was not registered in a prospective protocol database, consistent with its narrative design.

Literature Search

The literature search was conducted in PubMed/MEDLINE, Scopus, and Google Scholar and was designed around three conceptual domains: (1) T2DM and diabetes-related neuropathy; (2) auditory electrophysiology, including ABR, auditory brainstem evoked response, brainstem auditory evoked potentials, ABR, brainstem auditory evoked response (BAER), and brainstem evoked response audiometry (BERA); and (3) auditory pathway or neurological involvement, including central auditory pathway, auditory neuropathy, central nervous system, hearing dysfunction, and auditory pathway. These concepts were combined using Boolean operators and adapted to the indexing characteristics of each database. The search covered literature published approximately from 2000 through 2024. Reference lists of relevant retrieved articles and reviews were also manually screened for additional potentially eligible publications.

Eligibility Criteria

Studies were considered eligible if they (a) enrolled adults (≥18 years) with a diagnosis of T2DM; (b) reported ABR measurements, including at least one of wave I, III, or V absolute latencies, or interpeak intervals (I-III, III-V, I-V); (c) used a case-control, cross-sectional, or prospective observational design; and (d) provided sufficient methodological detail to interpret the electrophysiological findings. Studies exclusively involving type 1 diabetes, gestational diabetes, or paediatric populations were excluded unless they contained a separable adult T2DM subgroup that could be analysed independently. Editorials, conference abstracts, and non-peer-reviewed materials were not considered primary evidence, although they were occasionally consulted for background information.

Study Selection

Titles and abstracts were screened for relevance, after which potentially eligible full-text articles were assessed against the predefined eligibility criteria. Studies were included when they fulfilled the population, ABR outcome, study-design, and methodological-information requirements described in this review. Ten observational studies were ultimately included in the narrative synthesis. The review was not conducted using a prospectively maintained systematic-review screening database. Consequently, the exact numbers of records retrieved from each database, duplicate records removed, full-text articles excluded, and reasons for individual full-text exclusions were not systematically recorded during the original review process. These numerical data therefore cannot be reconstructed reliably retrospectively. Consequently, a PRISMA-style flow diagram was not generated. This limitation is explicitly acknowledged and represents a limitation of the reproducibility of the present narrative review.

Data Extraction

From each included study, the following information was extracted where available: first author, publication year, country, study design, sample size (T2DM and control groups), participant demographics (age, sex), duration of diabetes, glycated haemoglobin (HbA1c) levels, hearing status, presence of peripheral neuropathy, relevant comorbidities (e.g., hypertension, dyslipidaemia), ABR stimulus type (click), intensity (dB nHL), repetition rate, recording electrode montage, and the specific ABR outcomes reported (wave I, III, V latencies; I-III, III-V, I-V interpeak intervals). Principal findings and any reported associations with clinical variables were also recorded. Data were interpreted according to the definitions and analytical approaches used in the original publications; no attempt was made to reanalyse individual patient data.

Methodological Quality Appraisal

Because this was a narrative review, no formal risk-of-bias score was assigned. Instead, we used a predefined qualitative framework to assess sample size, participant selection, group comparability, hearing status, peripheral neuropathy, relevant confounders, ABR acquisition parameters, outcome reporting, and statistical methods. These domains informed interpretation rather than producing a numerical quality score. Studies with clearer methodology and better control of confounders received greater interpretive consideration, while findings from studies with significant methodological limitations or incomplete reporting were interpreted with caution. This framework was applied consistently throughout the narrative synthesis.

Handling of Heterogeneity

Heterogeneity across studies was assessed qualitatively, focusing on differences in participant characteristics (age, diabetes duration, HbA1c, comorbidities), ABR stimulus and recording parameters, outcome definitions, and statistical approaches. These factors were explicitly considered when comparing study findings. The presence of heterogeneity was a primary reason for not undertaking quantitative pooling; a meta-analytic approach was deemed inappropriate given the diversity of study designs, populations, and methodological details.

Narrative Synthesis

When findings differed between studies, they were not weighted solely according to the direction or statistical significance of an individual result. Greater interpretive weight was given to findings that were reproduced across multiple independent studies with broadly comparable participant characteristics and ABR methodology. Findings reported in only one or a small number of studies, or those arising from studies with substantial differences in participant selection, hearing-status assessment, ABR acquisition parameters, or control of potential confounding factors, were interpreted more cautiously. Where conflicting results were identified, the disagreement was retained in the synthesis and possible methodological and clinical explanations were considered rather than assigning a definitive preference to one finding.

Associations between ABR parameters and clinical variables, including HbA1c and diabetes duration, were interpreted based on the consistency of the reported association, its statistical significance where available, and whether relevant confounding factors were considered or adjusted for in the original study. Because the included evidence was predominantly cross-sectional, these associations were interpreted as observational relationships, not evidence of causality. Accordingly, consistent findings across several studies were considered suggestive of an underlying association, whereas inconsistent, sparsely reported, or inadequately adjusted findings were considered insufficient to support a firm conclusion.

The synthesis was conducted narratively, comparing findings across studies according to the following domains: (1) absolute wave latencies (waves I, III, V); (2) interpeak intervals (I-III, III-V, I-V); (3) patterns suggesting predominantly central versus peripheral involvement; (4) associations with HbA1c and diabetes duration; (5) relationships with other metabolic and demographic factors; and (6) methodological strengths and limitations. The review also integrated background literature on pathophysiological mechanisms and ABR neurophysiology to provide a contextual framework for interpreting the electrophysiological findings.

Crucially, the synthesis distinguishes between consistency in the direction of findings and the strength of evidence. The presence of multiple studies reporting similar associations does not constitute proof of causality; rather, it suggests a pattern that warrants further investigation. ABR abnormalities were interpreted as electrophysiological correlates that may be consistent with central auditory conduction delay, but they were not treated as definitive anatomical localisation of a specific lesion. A preserved wave I with prolonged later waves and interpeak intervals may suggest predominantly central dysfunction, whereas wave I prolongation may indicate additional peripheral auditory nerve involvement. However, ABR waves arise from distributed neural generators and can be influenced by peripheral hearing status, stimulus parameters, and recording techniques; thus, ABR findings alone cannot establish a discrete histopathological lesion [7,8].

Consideration of Confounding

Potential confounders, including age, sex, hearing impairment, hypertension, dyslipidaemia, smoking, obesity, peripheral neuropathy, renal disease, and diabetes duration, were not consistently adjusted for across the included studies. Therefore, the observed associations between ABR abnormalities and T2DM or glycaemic control should be interpreted with caution. The narrative synthesis explicitly acknowledges that unmeasured or inadequately controlled confounding may have influenced the reported findings, and the available evidence does not permit confident causal inferences.

Reporting Guidance

The review was conducted in accordance with the principles of the Scale for the Assessment of Narrative Review Articles (SANRA) to enhance transparency and methodological rigour [14]. However, because this is a narrative review, the reporting does not follow PRISMA or other systematic-review checklists.

Results

Characteristics of the Evidence Base

Ten observational studies met the eligibility criteria for this narrative review [10,12,15-22]. All employed case-control or cross-sectional designs, with the diabetic groups ranging from 30 to 200 participants and control groups of comparable size. The studies originated predominantly from India [10,12,15-18,20], with additional contributions from Indonesia [21] and Iran [22]. Collectively, the included investigations enrolled approximately 1,100 adults with T2DM and 800 non-diabetic controls, though precise aggregate totals are not reported in all studies.

Clinical characteristics varied considerably across cohorts. Diabetes duration ranged from newly diagnosed to more than 15 years, and glycated haemoglobin (HbA1c) levels spanned from 6.5% to 12.0%. Hearing status was assessed in most studies using pure-tone audiometry, although the criteria for normal hearing and the frequencies tested were not uniformly specified. The presence of peripheral neuropathy was documented in some cohorts but was not systematically evaluated or excluded in all investigations. Other comorbidities, including hypertension and dyslipidaemia, were reported inconsistently, limiting cross-study comparability.

All studies recorded click-evoked ABRs using surface electrodes. Stimulus intensities ranged from 70 to 90 dB nHL, and repetition rates varied between 11.1 and 21.1 clicks per second. Despite these methodological differences, each study reported absolute wave latencies and interpeak intervals as primary outcome measures, permitting qualitative comparison of conduction timing indices. Table 1 summarises the characteristics of the included studies.

**Table 1:** Characteristics of clinical studies evaluating auditory brainstem responses in adults with type 2 diabetes mellitus T2DM, type 2 diabetes mellitus; ABR, auditory brainstem response; HbA1c, glycated haemoglobin; NR, not reported; nHL, normal hearing level

Study	Country	Design	T2DM / Control	Key clinical characteristics	ABR methodology	Principal finding
Suresh et al., 2018 [10]	India	Case-control	60 / 60	Duration 3–12 yr; HbA1c 7.0–11.5%	Click, 90 dB nHL, 11.1/s	Prolonged III, V; I–III, III–V, I–V prolonged; wave I normal
Goyal et al., 2019 [15]	India	Case-control	50 / 50	Duration 5–15 yr; HbA1c 6.8–11.2%	Click, 80 dB nHL, 11.1/s	Prolonged III, V; I–III, III–V, I–V prolonged; wave I normal; association with HbA1c
Baweja et al., 2013 [12]	India	Case-control	50 / 50	Duration 2–10 yr; HbA1c 7.2–12.0%	Click, 80 dB nHL, 11.1/s	Prolonged I, III, V; all interpeak intervals prolonged
Mishra et al., 2023 [16]	India	Case-control	60 / 60	Duration 1–15 yr; HbA1c 6.5–10.8%	Click, 80 dB nHL, 11.1/s	Prolonged III, V; I–III, I–V prolonged; wave I normal
Batham et al., 2017 [17]	India	Cross-sectional	100 / 100	Duration 2–12 yr; HbA1c NR	Click, 80 dB nHL, 11.1/s	Prolonged III, V; III–V, I–V prolonged; wave I normal
Taruna and Zakiuddin, 2019 [18]	India	Case-control	50 / 50	Duration 3–10 yr; HbA1c 6.9–11.0%	Click, 80 dB nHL, 11.1/s	Prolonged III, V; I–V prolonged; wave I normal
Gupta et al., 2018 [19]	India	Case-control	60 / 60	Duration 2–14 yr; HbA1c 7.2–11.8%	Click, 90 dB nHL, 11.1/s	Prolonged III, V; I–III, I–V prolonged; wave I normal; association with duration and HbA1c
Samatra et al., 2020 [21]	Indonesia	Cross-sectional	40 / 30	Duration 1–10 yr; HbA1c NR	Click, 80 dB nHL, 11.1/s	Prolonged I, III, V in subgroup; interpeak intervals prolonged
Siddiqi et al., 2013 [20]	India	Case-control	60 / 60	Duration 2–8 yr; HbA1c 7.0–11.5%	Click, 80 dB nHL, 21.1/s	Prolonged I, III, V; all interpeak intervals prolonged
Sheikhzadeh et al., 2024 [22]	Iran	Case-control	30 / 30	Duration 1–6 yr; HbA1c 6.9–10.4%	Click, 80 dB nHL, 11.1/s	Prolonged III, V; I–V prolonged; wave I normal

Across the included studies, the most prominent methodological differences related to participant selection. Several investigations explicitly excluded individuals with hearing impairment or established peripheral neuropathy [10,15,18,22], while others did not specify such exclusions [12,17,21]. The duration of diabetes was reported continuously in some studies but was categorised in others, and HbA1c was either analysed as a continuous variable, dichotomised at a threshold (e.g., 7.0% or 8.0%), or not reported at all [17,21]. These variations in participant characterisation and outcome definition complicate direct comparison and may contribute to the inconsistent findings across studies.

ABR Findings in Adults With T2DM

Wave I latency: Wave I latency was reported as within normal limits in seven of the 10 included studies [10,15-19,22]. This finding suggests that distal auditory nerve function was relatively preserved in the majority of assessed cohorts. However, three studies reported significant prolongation of wave I latency in diabetic participants compared with controls [12,20,21]. In the study by Samatra et al., wave I prolongation was observed in a subgroup of patients with longer diabetes duration, but was not evident across the entire diabetic cohort [21]. Baweja et al. and Siddiqi et al. demonstrated wave I prolongation in their full diabetic groups, indicating that peripheral auditory nerve involvement may occur in some patient populations [12,20]. Thus, wave I abnormalities are not universal in T2DM; rather, they appear to be present in a subset of individuals, potentially reflecting more advanced disease, poorer glycaemic control, or concurrent peripheral neuropathy.

Wave III latency: Wave III latency was prolonged in all 10 studies when compared with control groups [10,12,15-22]. The magnitude of prolongation varied across studies, with mean delays ranging from approximately 0.15 to 0.30 ms relative to controls. This consistency across investigations is notable and suggests that slowed neural conduction at the level of the lower brainstem-encompassing the cochlear nucleus and superior olivary complex-represents a reproducible electrophysiological finding in adults with T2DM. However, the precise anatomical localisation of wave III remains an area of ongoing investigation; it is more accurately described as reflecting activity arising from distributed generators in the lower pons rather than from a single discrete nucleus.

Wave V latency: Wave V latency was also prolonged in all 10 included studies [10,12,15-22]. In many cohorts, the magnitude of wave V prolongation exceeded that of wave III, suggesting that conduction delay may accumulate as the neural signal ascends through the brainstem. The wave V latency abnormality was particularly prominent in studies that also reported normal wave I latencies [10,15-19,22], a pattern that is compatible with a predominantly central conduction deficit. Nevertheless, wave V prolongation alone does not establish a specific anatomical lesion; it may reflect altered synaptic transmission, demyelination, or axonal dysfunction at multiple sites along the auditory brainstem pathway.

Interpeak latencies: The I-III interpeak interval, reflecting conduction between the distal auditory nerve and lower brainstem, was prolonged in eight studies [10,12,15,16,19-21]. The III-V interval, representing conduction through more rostral brainstem segments, was prolonged in seven studies [10,12,15,17,19-21]. The I-V interval, a global measure of auditory brainstem conduction time, was prolonged in all 10 investigations [10,12,15-22]. In cohorts with normal wave I latencies, the selective prolongation of these interpeak intervals provides electrophysiological evidence compatible with central auditory conduction delay [10,15-19,22]. In the three studies where wave I was also prolonged, the magnitude of interpeak prolongation generally exceeded what would be expected from peripheral delay alone, suggesting that central dysfunction contributed even in these mixed-pattern cohorts [12,20,21]. Table 2 summarises the direction and consistency of ABR abnormalities across the included studies.

**Table 2:** Direction and consistency of auditory brainstem response abnormalities reported in adults with type 2 diabetes mellitus ↑ - significantly prolonged; ↔- no significant difference; NR- not reported

Study	Wave I	Wave III	Wave V	I–III	III–V	I–V
Suresh et al., 2018 [10]	↔	↑	↑	↑	↑	↑
Goyal et al., 2019 [15]	↔	↑	↑	↑	↑	↑
Baweja et al., 2013 [12]	↑	↑	↑	↑	↑	↑
Mishra et al., 2023 [16]	↔	↑	↑	↑	↔	↑
Batham et al., 2017 [17]	↔	↑	↑	NR	↑	↑
Taruna and Zakiuddin, 2019 [18]	↔	↑	↑	NR	NR	↑
Gupta et al., 2018 [19]	↔	↑	↑	↑	↔	↑
Samatra et al., 2020 [21]	↑ (subgroup)	↑	↑	↑	↑	↑
Siddiqi et al., 2013 [20]	↑	↑	↑	↑	↑	↑
Sheikhzadeh et al., 2024 [22]	↔	↑	↑	NR	NR	↑

The most consistently prolonged parameters across the evidence base were wave III latency, wave V latency, and the I-V interpeak interval. The I-III interval was also prolonged in the majority of studies, whereas the III-V interval showed less consistent prolongation, with several studies reporting either no significant difference or not reporting this parameter. This pattern may suggest that conduction slowing is most pronounced in the lower brainstem segment, although methodological differences in stimulus rate and recording techniques could influence the detectability of III-V prolongation.

Peripheral, Central, and Mixed ABR Patterns

The studies can be broadly categorised according to the pattern of ABR abnormalities observed. Seven investigations demonstrated a predominantly central-compatible pattern, characterised by normal wave I latency with prolongation of wave III, wave V, and interpeak intervals [10,15-19,22]. This pattern is electrophysiologically compatible with altered conduction within the auditory brainstem while preserving distal auditory nerve function. Three studies exhibited a mixed peripheral-central pattern, with both wave I prolongation and later-wave/interpeak abnormalities [12,20,21]. In these cohorts, the presence of wave I prolongation suggests concurrent auditory nerve involvement, while the interpeak prolongations indicate additional central conduction delay. No study reported isolated peripheral involvement without evidence of central conduction abnormality.

It is important to note that these classifications are based on electrophysiological patterns and should not be interpreted as definitive anatomical localisation. ABR waves arise from distributed neural generators and can be influenced by stimulus parameters, recording techniques, and peripheral hearing status. The relative preservation of wave I does not completely exclude peripheral auditory pathology, and wave prolongations do not prove a specific brainstem lesion. Nevertheless, the pattern observed across the majority of studies-preserved wave I with prolonged later waves and interpeak intervals-is compatible with the hypothesis that central auditory pathway dysfunction is a prominent feature of diabetes-related neural injury in at least a substantial proportion of patients.

Association With Glycaemic Control and Diabetes Duration

Glycaemic control (HbA1c): Several studies examined the relationship between HbA1c and ABR parameters. Goyal et al. reported significant positive correlations between HbA1c and wave III latency, wave V latency, and the I-III and I-V interpeak intervals [15]. Mishra et al. observed similar associations, with higher HbA1c levels corresponding to greater prolongation of wave V and the I-V interval [16]. Gupta et al. demonstrated correlations between HbA1c and both wave III latency and the I-V interval [19]. Sheikhzadeh et al. reported a significant association between HbA1c and wave V latency in their cohort [22]. However, two studies that stratified participants by glycaemic control found only non-significant trends [17,21], and others did not perform formal correlation analyses. Thus, although the direction of association was generally consistent across studies that examined it, the strength and statistical significance varied, and the evidence should be considered suggestive rather than definitive.

Diabetes duration: Longer diabetes duration was associated with more pronounced ABR abnormalities in the majority of studies that evaluated this variable. Suresh et al. reported greater prolongation of wave V and the I-V interval in patients with diabetes duration exceeding 5 years [10]. Baweja et al. observed that patients with duration greater than 10 years had significantly longer wave latencies and interpeak intervals than those with shorter duration [12]. Mishra et al. and Gupta et al. both reported positive correlations between diabetes duration and wave V latency [16,19]. Siddiqi et al. noted that duration was associated with the degree of I-V prolongation [20]. However, two studies did not detect an independent effect of diabetes duration after adjusting for HbA1c [18,22], and another did not report duration-related analyses [21]. Therefore, while a relationship between longer diabetes duration and greater ABR abnormality appears to exist in many cohorts, the interdependence of duration and glycaemic control complicates interpretation of their relative contributions.

Other Clinical and Metabolic Variables

Evidence regarding other clinical and metabolic variables was sparse and inconsistent. Age was examined in several studies, but no consistent independent association with ABR latencies emerged after adjustment for diabetes duration and glycaemic control. Body mass index showed weak or non-significant correlations in the few studies that reported it [19,21]. Smoking status was examined only in one study, which noted a non-significant trend [19]. Lipid profile parameters were also evaluated only sporadically, with inconsistent results. The limited and heterogeneous data on these variables preclude definitive conclusions regarding their independent contributions to ABR abnormalities in T2DM. Table 3 demonstrates clinical and metabolic variables associated with ABR abnormalities in adults with T2DM.

**Table 3:** Clinical and metabolic variables associated with ABR abnormalities in adults with type 2 diabetes mellitus References in square brackets indicate the studies that reported the corresponding association; multiple references separated by commas denote studies with consistent findings, while those in parentheses indicate studies with non‑significant or conflicting results. ABR: auditory brainstem response; HbA1c: glycated haemoglobin; NR: not reported

Variable	Direction of association	ABR parameters involved	Supporting studies (references)	Consistency across studies	Interpretation
HbA1c	Positive	III, V; I–III, I–V	[15,16,19,22] (significant); [17,21] (trend only)	Moderately consistent	Suggestive of association, but not uniform
Diabetes duration	Positive	V; I–V	[10,12,16,19,20] (significant); [18,22] (not independent)	Moderately consistent	May be confounded by HbA1c
Age	Weak/inconsistent	III, V	[15,19] (weak or no association)	Limited	No consistent independent effect
Body mass index	Weak	III, V	[19,21] (weak); [15,16] (none)	Limited	Insufficient evidence
Smoking	Inconsistent	NR	[19] (trend only)	Very limited	Insufficient evidence
Lipid profile	Inconsistent	NR	[19]	Very limited	Insufficient evidence
Peripheral neuropathy	Positive	III, V; I–V	[18]	Limited	Limited evidence, but plausible

Associations were interpreted primarily according to the consistency and reproducibility of findings across studies, with consideration given to differences in study populations, ABR methodology, statistical analysis, and adjustment for potential confounders. Findings consistently reported across multiple studies were regarded as more informative than isolated statistically significant associations. By contrast, discordant or sparsely reported findings were deemed insufficient to support firm conclusions.

Peripheral neuropathy was assessed in some cohorts, and its presence was generally associated with greater ABR prolongation. However, only one study specifically compared ABR parameters in T2DM patients with and without peripheral neuropathy [18], and the findings suggested that central auditory dysfunction may occur independently of peripheral neuropathy. This observation warrants further investigation but is insufficient to establish a definitive conclusion.

Overall Evidence Pattern

The collective evidence from the 10 reviewed studies demonstrates that adults with T2DM frequently exhibit ABR abnormalities, particularly prolongation of wave III, wave V, and interpeak intervals. The most reproducible finding across the evidence base is prolongation of the I-V interpeak interval, which was reported in all 10 studies. Wave III and wave V latencies were also consistently prolonged, although the magnitude of delay varied. Wave I was preserved in the majority of cohorts, suggesting that peripheral auditory nerve function may remain intact in many patients, while central conduction abnormalities are more consistently observed.

However, the evidence is not uniform. Three studies reported wave I prolongation, indicating that peripheral auditory nerve involvement occurs in a subset of patients. The associations with HbA1c and diabetes duration were generally positive but not uniformly statistically significant, and the cross-sectional design of all included studies precludes causal inference. Methodological heterogeneity, including differences in participant selection, hearing-status assessment, ABR acquisition protocols, and statistical approaches, limits direct comparison across studies and may account for some of the observed inconsistencies.

The pattern of ABR abnormalities is electrophysiologically compatible with altered central auditory conduction in a substantial proportion of adults with T2DM. However, the available evidence does not establish that these abnormalities definitively represent central diabetic neuropathy, nor does it demonstrate that ABR can reliably distinguish central from peripheral auditory dysfunction. The findings are suggestive but require confirmation through larger, methodologically standardised, and preferably longitudinal investigations.

Discussion

The available evidence demonstrates that adults with T2DM frequently exhibit abnormalities in ABR parameters, with prolonged wave III and wave V latencies and extended interpeak intervals representing the most reproducible electrophysiological findings. The I-V interpeak interval was prolonged in all 10 reviewed studies, suggesting that altered auditory brainstem conduction timing is a consistent feature across diverse clinical cohorts. Wave III and wave V latencies were also prolonged in every investigation, although the magnitude of prolongation varied considerably. By contrast, wave I latency was preserved in seven of the 10 studies, indicating that distal auditory nerve function may remain relatively intact in many patients, while central conduction abnormalities appear more consistently.

Wave III and wave V reflect neural activity generated by distributed sources within the auditory brainstem, rather than by single discrete anatomical structures [7,8]. Prolongation of these waves, especially when wave I remains within the expected range, is consistent with delayed central auditory conduction. The I-III and I-V interpeak intervals offer further insight into conduction timing along the auditory pathway. However, ABR findings should be regarded as electrophysiological indicators and not as definitive evidence for the anatomical localisation of a specific lesion. Their prolongation, particularly when wave I is normal, suggests that conduction slowing occurs at sites central to the auditory nerve. The prolongation of the I-III interval, observed in eight studies, further localises the initial conduction delay to the caudal brainstem. However, it is important to emphasise that ABR waveforms do not arise from single discrete anatomical structures; they reflect summed neural activity from multiple generators, and their precise localisation remains inferential [8]. The observed pattern is therefore compatible with central auditory conduction dysfunction but does not establish a specific histopathological lesion.

The three studies that reported wave I prolongation indicate that peripheral auditory nerve involvement can occur in a subset of patients [12,20,21]. This mixed peripheral-central pattern may reflect more advanced disease, poorer glycaemic control, or concurrent peripheral neuropathy affecting the cochlear nerve. Importantly, no study reported isolated peripheral involvement without concomitant central abnormalities, reinforcing the concept that brainstem auditory pathways are a principal site of diabetes-related neural injury. Nevertheless, the presence of mixed patterns underscores that auditory dysfunction in T2DM is not exclusively central; rather, it exists along a spectrum that may include peripheral, central, or combined involvement depending on individual patient characteristics.

The relationship between ABR abnormalities and glycaemic control warrants cautious interpretation. Several studies reported positive associations between HbA1c and prolonged wave III, wave V, and interpeak latencies [15,16,19,22], suggesting that greater hyperglycaemic exposure may be associated with more pronounced conduction delay. However, this relationship was not statistically significant in all cohorts [17,21], and the cross-sectional design of all included studies prevents causal inference. Single HbA1c measurements may not adequately reflect cumulative glycaemic exposure over years, and residual confounding from unmeasured variables-including hypertension, dyslipidaemia, and genetic susceptibility-cannot be excluded. Diabetes duration showed a similar pattern: longer duration was associated with greater ABR abnormalities in most studies [10,12,16,19,20], but the independent contribution of duration was difficult to separate from HbA1c because the two variables are intrinsically correlated [18,22].

The auditory brainstem nuclei, with their high metabolic demands and sustained firing rates, may be particularly vulnerable to these insults. Experimental studies have demonstrated diabetes-related structural and molecular abnormalities within auditory structures, including changes associated with oxidative stress and mitochondrial dysfunction [15,16]. However, these findings should be interpreted cautiously because much of the mechanistic evidence derives from experimental models or peripheral auditory structures rather than direct examination of the human auditory brainstem. Consequently, these mechanisms provide biological plausibility for the observed ABR abnormalities but do not establish the precise pathological substrate responsible for delayed central auditory conduction in humans.

From a clinical perspective, the presence of ABR abnormalities in patients with normal pure-tone audiometry suggests that central auditory dysfunction may develop before clinically detectable hearing loss [15,17,22]. This observation raises the possibility that ABR could serve as a subclinical electrophysiological marker of central neural involvement in T2DM. However, the current evidence does not establish ABR as a validated screening or prognostic biomarker. Critical gaps remain: sensitivity, specificity, diagnostic thresholds, reproducibility across laboratories, and prognostic value have not been adequately characterised. Furthermore, the predominantly cross-sectional design of available studies precludes determination of whether ABR abnormalities precede or follow clinically meaningful auditory or neurological outcomes. Routine clinical ABR screening in asymptomatic individuals with T2DM is therefore not supported by the available evidence.

Methodological heterogeneity represents a major limitation of the existing literature and constrains the strength of conclusions that can be drawn. Included studies differed substantially in participant selection, including age distributions, diabetes duration, glycaemic control, hearing status, presence of peripheral neuropathy, and prevalence of comorbidities such as hypertension and dyslipidaemia. ABR acquisition protocols varied in stimulus intensity (70-90 dB nHL), repetition rate (11.1-21.1/s), and electrode montage, all of which can influence latency measurements. Sample sizes were generally modest, ranging from 30 to 200 diabetic participants, and statistical adjustment for potential confounders was inconsistent. The geographic concentration of studies in India, while providing a substantial evidence base, limits external generalisability to other ethnic populations and healthcare settings. These factors likely contribute to some of the observed inconsistencies and should be considered when interpreting the collective findings.

This narrative review has several strengths. It provides a clinically focused synthesis of ABR evidence in T2DM, integrates electrophysiological findings with clinical variables, distinguishes between peripheral and central-compatible patterns, and explicitly acknowledges the methodological limitations and remaining uncertainties in the literature. However, the review itself has limitations. As a narrative review, it does not employ systematic search methodology or quantitative pooling, and the absence of a formal risk-of-bias assessment limits the ability to grade the quality of individual studies. The search was not prospectively registered, and the possibility of publication or language bias cannot be excluded. Additionally, the number of included studies is relatively small, and the evidence base is predominantly derived from single-centre observational investigations.

Future research should prioritise multicentre prospective studies employing standardised ABR protocols and comprehensive metabolic and auditory phenotyping. Longitudinal designs are essential to determine whether ABR abnormalities progress over time and whether they predict subsequent hearing loss, cognitive decline, or other neurological outcomes. Intervention studies examining the effect of intensive glycaemic control or other metabolic interventions on ABR parameters would provide valuable insights into causality and potential reversibility. Multimodal investigations combining ABR with other evoked potentials, neuroimaging, and cognitive assessments could map the full extent of central sensory pathway involvement in T2DM and clarify the relationship between electrophysiological abnormalities and clinically meaningful outcomes.

## Conclusions

The available evidence indicates that adults with T2DM frequently exhibit electrophysiological evidence of altered auditory brainstem conduction, characterised most reproducibly by prolongation of wave III, wave V, and the I-V interpeak interval. In the majority of cohorts, wave I latency remains preserved, a pattern compatible with predominantly central conduction dysfunction, although mixed peripheral-central abnormalities occur in a subset of patients. Associations with glycaemic control and diabetes duration are suggestive but not uniformly demonstrated, and the cross-sectional nature of the evidence precludes causal inference.

This narrative review provides a critical synthesis of current knowledge, distinguishes between central and peripheral patterns, and highlights the methodological heterogeneity and unresolved questions that limit clinical translation. The available evidence does not support routine ABR screening for subclinical central diabetic neuropathy in clinical practice, but it does identify ABR as a promising electrophysiological tool requiring further investigation. Well-designed, multicentre, longitudinal studies with standardised protocols are needed to validate these findings, establish prognostic value, and determine whether ABR abnormalities represent a modifiable target for metabolic intervention.
